# Akting up in the GABA hypothesis of schizophrenia: Akt1 deficiency modulates GABAergic functions and hippocampus-dependent functions

**DOI:** 10.1038/srep33095

**Published:** 2016-09-12

**Authors:** Chia-Yuan Chang, Yi-Wen Chen, Tsu-Wei Wang, Wen-Sung Lai

**Affiliations:** 1 Department of Psychology, National Taiwan University, Taipei, Taiwan; 2 Department of Life Science, National Taiwan Normal University, Taipei, Taiwan; 3 Graduate Institute of Brain and Mind Sciences, National Taiwan University, Taipei, Taiwan; 4 Neurobiology and Cognitive Science Center, National Taiwan University, Taipei, Taiwan

## Abstract

Accumulating evidence implies that both *AKT1* and *GABA*_*A*_ receptor (*GABA*_*A*_*R*) subunit genes are involved in schizophrenia pathogenesis. Activated Akt promotes GABAergic neuron differentiation and increases GABA_A_R expression on the plasma membrane. To elucidate the role of Akt1 in modulating GABAergic functions and schizophrenia-related cognitive deficits, a set of 6 *in vitro* and *in vivo* experiments was conducted. First, an Akt1/2 inhibitor was applied to evaluate its effect on GABAergic neuron-like cell formation from P19 cells. Inhibiting Akt resulted in a reduction in parvalbumin-positive neuron-like cells. In *Akt1*^−/−^ and wild-type mice, seizures induced using pentylenetetrazol (a GABA_A_R antagonist) were measured, and GABA_A_R expression and GABAergic interneuron abundance in the brain were examined. Female *Akt1*^−/−^ mice, but not male *Akt1*^−/−^ mice, exhibited less pentylenetetrazol-induced convulsive activity than their corresponding wild-type controls. Reduced parvalbumin-positive interneuron abundance and GABA_A_R subunit expression, especially in the hippocampus, were also observed in female *Akt1*^−/−^ mice compared to female wild-type mice. Neuromorphometric analyses revealed significantly reduced neurite complexity in hippocampal pyramidal neurons. Additionally, female *Akt1*^−/−^ mice displayed increased hippocampal oscillation power and impaired spatial memory compared to female wild-type mice. Our findings suggest that *Akt1* deficiency modulates GABAergic interneurons and GABA_A_R expression, contributing to hippocampus-dependent cognitive functional impairment.

The Akt (also known as protein kinase B) family consists of three serine/threonine kinases (Akt1, Akt2, and Akt3) that are involved in multiple biological functions and cellular processes. The Akt family is highly conserved in mammals, and the amino acid sequences of mouse, rat and human Akts share approximately 95% identity^1^. Recent advances in genome-wide analyses indicated that there might be a common pathophysiology among several psychiatric disorders^2^ and that deregulation of Akt signaling pathways is directly associated with some of the most prevalent and incurable human disorders, including schizophrenia^3^. Accumulating evidence also suggests that multiple susceptibility genes, including *Akt1* (*PKBα*)^4^ and GABA_A_ receptor (GABA_A_R) subunit genes^5^,^6^, might contribute to the pathogenesis of schizophrenia. Evidence supporting *Akt1* as a susceptibility gene for schizophrenia was originally reported in Caucasian families of European descent and was subsequently confirmed in several other ethnic groups^4^,^7^,^8^. Studies of the postmortem brains of schizophrenia patients^4^,^9^ and *Akt1*-deficient mice^10^,^11^,^12^,^13^,^14^ as well as functional neuroimaging analyses in humans^15^,^16^ support the concept that variations in the *Akt1* gene or its encoded protein have epistatic effects on dopamine-related functions. The biological functions of Akt1 and the mechanism by which Akt1 contributes to susceptibility for schizophrenia are currently under intensive investigation.

Schizophrenia is a multifactorial disorder for which there is a strong genetic predisposition, but the exact cause of schizophrenia remains unclear. Complementary to the long-standing dopamine and glutamate hypotheses of schizophrenia, GABA, the primary inhibitory neurotransmitter in the brain, has attracted increasing attention in the search for the neural mechanisms underlying the cognitive deficits observed in schizophrenia^17^,^18^. Evidence supporting the role of GABA in schizophrenia development includes the deficiency of GABA synthesis resulting from reduced transcription of the 67-kDa isoform of glutamic acid decarboxylase (GAD67) within parvalbumin-immunoreactive cortical neurons and the reduction in the subpopulation of GABAergic interneurons positive for parvalbumin and the β2 subunit of the GABA_A_ receptor (GABA_A_R) in animal studies and in the post-mortem brains of schizophrenia patients^18^,^19^,^20^,^21^,^22^. More specifically, cognitive dysfunction is considered a core feature of schizophrenia that not only strongly influences the quality of life and functionality of people with this illness^23^,^24^ but also predicts the long-term functional outcome of these patients^25^. The GABA hypothesis further predicts that abnormalities in GABAergic neurons lead to impairments in cortical gamma synchrony and corresponding cognitive control in schizophrenia^26^. Indeed, schizophrenic patients exhibited deficits in gamma oscillations^27^, and this finding suggests that altered GABA signalling may associate dysfunction of gamma activity with the pathology of schizophrenia.

Intriguingly, emerging *in vitro* studies indicated the involvement of Akt signalling in the regulation of GABAergic synapses. It was reported that the activation of Akt signalling facilitated GABA_A_R trafficking to the membrane via the phosphorylation of β2 GABA_A_R subunits^28^. Active PDK1-Akt signalling can promote the differentiation of telencephalic neural precursor cells into GABAergic, but not glutamatergic, neurons^29^. It was also reported that the regulation of hippocampal neurogenesis by the protein Disrupted-in-Schizophrenia 1 requires GABA-induced depolarization through a convergence onto Akt- mammalian target of rapamycin (mTOR) signalling pathway^30^. Similarly, our recent findings further revealed reductions in the morphological complexity and alterations in the electrophysiological properties of striatal GABAergic medium spiny neurons in homozygous *Akt1*-knockout (*Akt1*^−/−^) mice^12^. It is of great interest to investigate how Akt1 regulates GABA signalling and GABA-related cognitive functions that might contribute to the pathogenesis of schizophrenia and its associated cognitive deficits.

As a complement to human studies, animal models provide an indispensable and practical approach to elucidate causal relationships between genetic deficits and functions, especially in genetically modified mice. Distinct sex-specific phenotypes were identified in *Akt1*-deficient mice^11^,^12^, and the Akt1 isoform was reported to be involved in the regulation of hippocampal neuroplasticity and cognition^31^; this regulatory activity of Akt1 might contribute to the pathogenesis of schizophrenia. Given the importance of Akt1 signalling and GABAergic function in the pathogenesis of schizophrenia and the involvement of Akt1 in the regulation of hippocampal functions, the primary goal of this study was to evaluate whether Akt1 deficiency causes any alteration in GABAergic function or hippocampus-related function at multiple levels. A set of 6 experiments was designed to identify the role of Akt1 in GABAergic signalling and related cognitive functions *in vitro* and *in vivo*. Our findings indicated that *Akt1* deficiency modulates the number of GABAergic interneurons, GABA_A_R expression, and neuronal morphology in the hippocampus. These effects of *Akt1* deficiency might contribute to the impairment of neuronal oscillations and of hippocampus-dependent cognitive functions in female *Akt1*^−/−^ mice.

## Results

### Experiment 1: Akt1/2 inhibitor application disrupted the differentiation of P19 cells into GABAergic neuron-like cells

**Figure 1:**
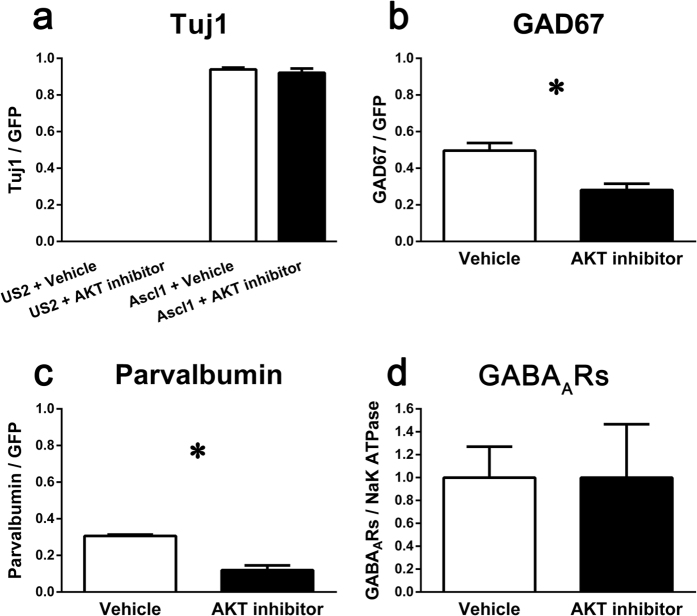
Experiment 1: Inhibiting Akt1/2 had no effect on overall neuronal differentiation but resulted in the proportion of significantly fewer GABAergic neuron-like cells from P19 mouse embryonal carcinoma cells. (**a**) Akt1/2 inhibitor application had no effect on the proportion of differentiated neuron-like cells (Tuj1/GFP, mean ± SEM) derived from P19 cells (Tuj1, neuronal marker; GFP, transfection marker; US2, control plasmid for transfection; Ascl1 (Mash1), inducer of neuronal differentiation; Vehicle, vehicle control; Akt inhibitor, Akt1/2 inhibitor). (**b**) Akt1/2 inhibitor application resulted in a significant reduction of the proportion of GABAergic neuron-like cells (GAD67/GFP, mean ± SEM) derived from P19 cells (GAD67, GABA marker; GFP, transfection marker). (**c**) Akt1/2 inhibitor application resulted in a significant reduction in the proportion of parvalbumin-positive neuron-like cells (parvalbumin/GFP, mean ± SEM) derived from P19 cells (GFP, transfection marker). (**d**) Treatment with the Akt1/2 inhibitor had no significant effect on the proportion of cells with GABA_A_R expression on the plasma membrane (GABA_A_Rs/Na^+^/K^+^ ATPase, mean ± SEM) (Na^+^/K^+^ ATPase, loading control for membrane proteins). *p < 0.05.

An Akt1/2 inhibitor was used to evaluate the effect of Akt deficiency on the differentiation of P19 mouse embryonal carcinoma cells (P19 cells) into GABAergic neuron-like cells which were reported to express glutamic acid decarboxylase and multiple GABA_A_R subunits. As shown in Fig. 1a, Akt1/2 inhibitor application did not affect the Ascl1-induced differentiation of P19 cells into neuron-like cells; a representative image is shown in Figure S1. In contrast, Akt1/2 inhibitor application resulted in a 40% reduction in the proportion of GABAergic neuron-like cells (*t*(4) = 4.004, *p* < 0.05; Fig. 1b and Figure S2) and a 60% reduction in the proportion of parvalbumin-positive neuron-like cells (*t*(4)* = *6.990, *p* < 0.05; Fig. 1c and Figure S3) compared to vehicle treatment. However, Akt inhibitor treatment did not affect the expression of functional GABA_A_Rs, as measured by the expression of the GABA_A_R β2 subunit at plasma membrane (Fig. 1d). These results revealed that inhibition of Akt had no effect on overall neuronal differentiation but resulted in a significant reduction in the proportion of GABAergic neuron-like cells in a P19 cell culture model.

### Experiment 2: Pentylenetetrazol-induced convulsive activity is reduced in female *Akt1* ^−/−^ mice compared with female wild-type (WT) mice

**Figure 2:**
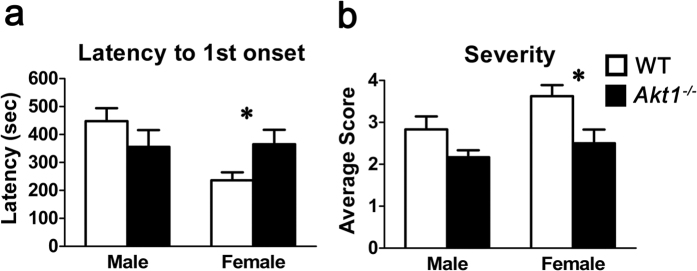
Experiment 2: Seizures induced by PTZ (a GABA_A_R antagonist and a CNS convulsant) in male and female *Akt1*^−/−^ mice and their WT littermate controls. Measurement of PTZ-induced seizures in WT control mice (white bar) and *Akt1*^−/−^ mice (black bar). (**a**) The latency to initial seizure onset (mean ± SEM, sec) for both males (n = 6 each; Left) and females (n = 8 each; Right). The female *Akt1*^−/−^ mice exhibited delayed seizure onset compared to the female WT mice. (**b**) The average seizure severity score (0–4) (mean ± SEM, sec) for both male (n = 6; Left) and female (n = 8; Right) mice. Seizure severity scores: 0, no signs of motor seizures; 1, isolated twitches; 2, tonic-clonic convulsions; 3, tonic extension; and 4, death. Female *Akt1*^−/−^ mice showed lower seizure severity scores than female WT mice. *p < 0.05.

As reduced Akt1/2 activity disrupted the differentiation of GABAergic neurons but did not alter overall neuronal differentiation *in vitro*, we sought to determine whether reducing Akt1 activity changed GABAergic function on a global scale *in vivo*. For this purpose, we induced seizures using pentylenetetrazol (PTZ), a GABA_A_ receptor antagonist and a CNS convulsant, in both male and female *Akt1*^−/−^ and WT mice. In female *Akt1*^−/−^ mice, the onset latency was significantly increased (*t*(14) = 2.177, *p* < 0.05; Fig. 2a) and seizure severity was decreased (*t*(14) = 3.12, *p* < 0.05; Fig. 2b) compared to female WT littermates. In contrast, no difference in either onset latency (*p* = 0.253) or seizure severity (*p* = 0.0856) was found between *Akt1*^−/−^ and WT males. Thus, Akt1 deficiency modulated the convulsive activity induced by a general GABA_A_R antagonist, especially in female mice.

### Experiment 3: Reduced density of parvalbumin-positive interneurons and GABA_A_R expression in the hippocampus of female *Akt1* ^−/−^ mice

**Figure 3:**
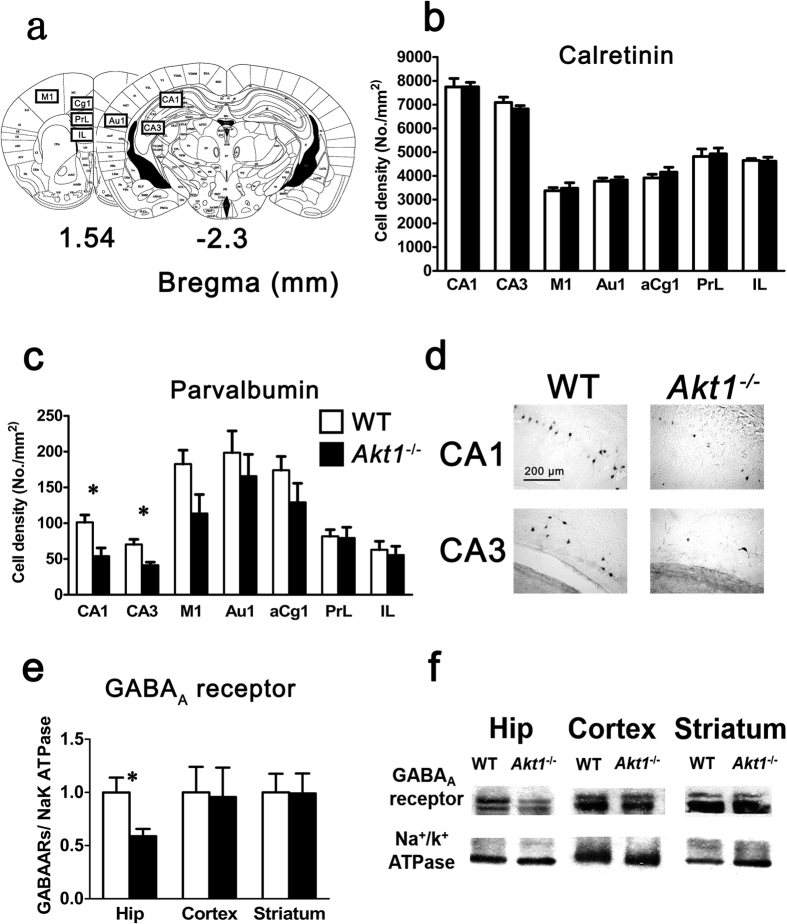
Experiment 3: Reduced numbers (cell density; cells/mm^2^; mean ± SEM) of parvalbumin-positive interneurons and reduced expression of GABA_A_ receptors were observed in the hippocampus of female *Akt1*^−/−^ mice (black bar; *n = *6) compared to female WT littermate controls (white bar; *n = *8). (**a**) Mouse brain atlases highlighting the regions of interest: hippocampal CA1 region (CA1), hippocampal CA3 region (CA3), primary motor cortex (M1), primary auditory cortex (Au1), anterior cingulate cortex, area 1 (aCg1), prelimbic cortex (PrL), and infralimbic cortex (IL). (**b**) Calretinin-positive interneurons in the different brain regions of female mice. (**c**) Parvalbumin-positive interneurons in the different brain regions of female mice. The cell density in the CA1 and CA3 areas was lower in female *Akt1*^−/−^ mice than in female WT mice. (**d**) Representative immunohistochemical images of parvalbumin-positive interneurons in the dorsal hippocampus (CA1 and CA3 areas; scale bar: 200 μm). (**e**) The expression of functional GABA_A_Rs in the hippocampus, cortex, and striatum of female mice. A significant difference in functional GABA_A_R expression in the hippocampus was found between *Akt1*^−/−^ and WT females. (**f**) Representative Western blot images of functional GABA_A_R expression (GABA_A_R: GABA_A_R β2 subunit; Na^+^/K^+^ ATPase: loading control for membrane proteins). *p < 0.05.

GABA plays an important role in mediating pre- and post-synaptic inhibition of neuronal activity. Because PTZ-induced seizures were suppressed in female *Akt1*^−/−^ mice, we examined whether the number of GABAergic interneurons (Experiment 3a) or the GABA_A_R expression level (Experiment 3b) in the target brain regions was altered in naïve female *Akt1*^−/−^ mice compared to female WT mice. Among all brain areas examined (Fig. 3a), immunohistochemistry revealed no genotypic difference in the abundance of calretinin-positive interneurons (Fig. 3b). In contrast, female *Akt1*^−/−^ mice showed significant decreases in the expression of parvalbumin-positive interneurons in the CA1 region (*t*(14) = 3.074, *p* < 0.05) and the CA3 region (*t*(14) = 3.557, *p* < 0.05) of the dorsal hippocampus (Fig. 3c,d). Western blot analysis revealed a significant reduction in the expression of the GABA_A_R β2 subunit in the hippocampus (*t*(8) = 2.652, *p* < 0.05), but not in the striatum or the cortex (Fig. 3e,f), of *Akt1*^−/−^ mice compared to WT mice. These results indicated that Akt1 deficiency contributed to a reduction in the density of parvalbumin-positive interneurons and in the expression of functional GABA_A_Rs in female mice, especially in the hippocampus.

### Experiment 4: Reduced neurite complexity of hippocampal CA1 pyramidal neurons in female *Akt1* ^−/−^ mice

**Figure 4:**
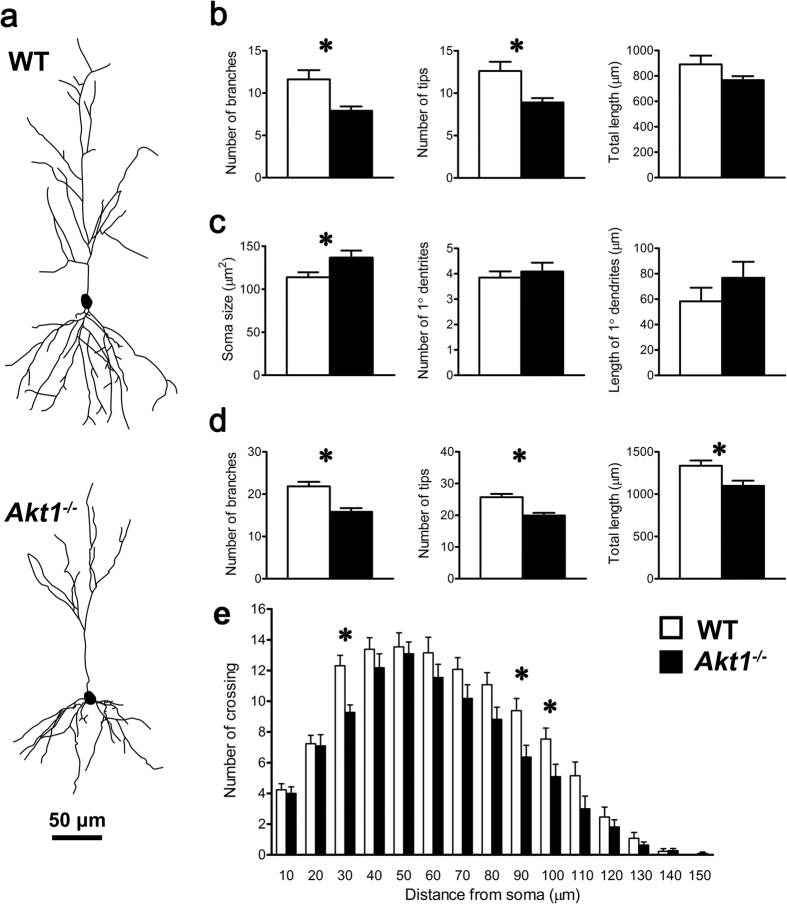
Experiment 4: Examination of the morphological features (mean ± SEM) of pyramidal neurons in the CA1 region of the dorsal hippocampus in female WT mice (n = 13; white bars) and female *Akt1*^−/−^ mice (n = 11; black bars). (**a**) Representative tracings of dorsal hippocampal pyramidal neurons from WT and *Akt1*^−/−^ mice (scale bar: 50 μm). (**b**) Apical neuronal properties (left to right: number of branches, number of tips, and total length of the apical tuft (μm)). (**c**) Basal neuronal properties (left to right: soma size (μm^2^), number of primary dendrites, and total length of primary dendrites (μm)). (**d**) Additional basal neuronal properties (left to right: number of branches, number of tips, and total length (μm)). (**e**) Sholl analysis of basal dendritic complexity within 10-μm concentric spheres around the soma. *p < 0.05.

The generation of seizures is controlled by the balance of network excitation and inhibition. Given that genetic deletion of Akt1 in mice resulted in a reduction in neuronal morphological complexity in the brain^10^,^11^,^12^ and that GABAergic interneurons primarily innervate the dendrites of hippocampal CA1 pyramidal cells and actively contribute to the input/output signalling of these neurons^32^,^33^, we determined the impact of Akt1 deficiency on the morphological characteristics of pyramidal neurons in the dorsal hippocampal CA1 region of Thy1-C57BL6-Tg (GFPm) transgenic mice. Representative tracings of GFP-labelled CA1 pyramidal neurons in the hippocampus of female WT and *Akt1*^−/−^ mice are shown in Fig. 4a. A quantitative evaluation of the GFP-labelled CA1 pyramidal neurons revealed significant morphological changes in the apical and basal dendritic architecture and its complexity. In apical dendrites, the numbers of dendritic branches (32%, *t*(22) = 2.887, *p* < 0.05; left panel of Fig. 4b) and tips (30%, *t*(22) = 2.887, *p* < 0.05; middle panel of Fig. 4b) were decreased in the *Akt1*^−/−^ females compared with the WT females. In basal dendrites, the soma size was significantly increased (20%, *t*(22) = 2.371, *p* < *0*.05; left panel of Fig. 4c), the number of branches (28%, *t*(22) = 4.269, *p* < *0*.05), the number of tips (22%, *t*(22) = 4.224, *p* < *0*.05), and the total dendrite length were reduced (18%, *t*(22) = 2.688, *p* < *0*.05) in the *Akt1*^−/−^ females compared to the WT females, as depicted in Fig. 4d. As shown in Fig. 4e, the effect of Akt deficiency on morphological complexity was confirmed via Sholl analysis (*F*(1, 22) = 7.003, *p* < *0*.05). No significant differences in other morphological variables were found. These results revealed significant reductions in the dendritic complexity of hippocampal CA1 pyramidal neurons in female *Akt1*^−/−^ mice that might affect neural activity and hippocampal output.

### Experiment 5: Alteration of hippocampal oscillations was detected in anaesthetized female *Akt1* ^−/−^ mice

**Figure 5:**
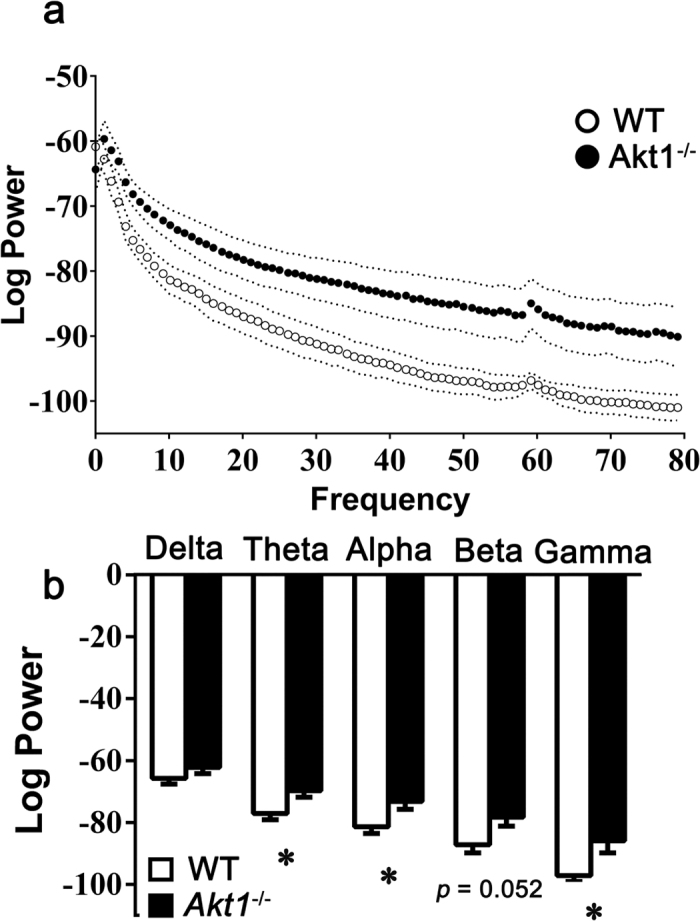
Experiment 5: Altered neural oscillations from the hippocampal CA1 region were identified in anaesthetized female *Akt1*^−/−^ mice compared to female WT littermate controls. (**a**) The power spectrum density (mean ± SEM) of hippocampal LFPs in the high frequency ranges was greater in anaesthetized female *Akt1*^−/−^ mice (white dot; *n = *5) than in female WT controls (black dot; *n = *5). Original data showing the log oscillation power from low frequency to high frequency (from left to right: 0–80 Hz; delta: 0–4 Hz; theta: 5–8 Hz; alpha: 9–12 Hz; beta: 12–30 Hz; and gamma: 30–80 Hz). (**b**) The average log oscillation power (mean ± SEM) in female *Akt1*^−/−^ mice (black bar) was greater than that in WT controls (white bar) in the high frequency ranges. *p < 0.05.

The balance between excitation and inhibition plays a critical role in hippocampal neural oscillation^34^. Based on the observed alterations in the hippocampus of female *Akt1*^−/−^ mice, local field potentials were recorded in the hippocampal CA1 region. The power spectrum density of the hippocampal local field potentials is shown in Fig. 5a. Compared to female WT mice, female *Akt1*^−/−^ mice displayed higher oscillation power in the theta (*t*(8) = 2.629, *p* < 0.05), alpha (*t*(8) = 2.497, *p* < 0.05), beta (*t*(8) = 2.286, *p* = 0.052), and gamma (*t*(8) = 2.585, *p* < 0.05) frequency ranges but not in the delta frequency range (*t*(8) = 1.293, *p = *0.232), as depicted in Fig. 5b. Alteration of neural oscillations in the CA1 region of the dorsal hippocampus in anaesthetized female *Akt1*^−/−^ mice was confirmed in this experiment.

### Experiment 6: Female *Akt1* ^−/−^ mice displayed impairments in hippocampus-related cognitive function

**Figure 6:**
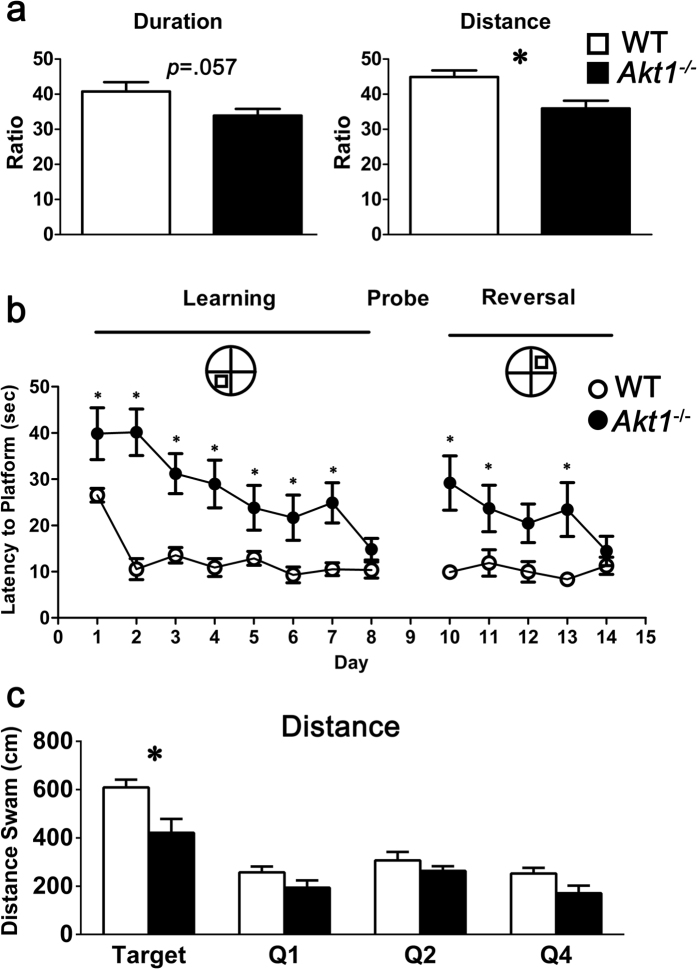
Female *Akt1*^−/−^ mice exhibited impaired hippocampus-related cognitive performance on the Y-maze (Experiment 6a) and Morris water maze tasks (Experiment 6b). (**a**) The percentages (%, mean ± SEM) of time spent and distance travelled in the novel arm of the Y-maze. Female *Akt1*^−/−^ mice (black bars; n = 8) exhibited reduced relative duration and distance in the novel arm of the Y-maze compared to female WT littermate controls (white bars; n = 8). (**b**) The escape latency (mean ± SEM sec) to reach the hidden platform in the Morris water maze task. Female *Akt1*^−/−^ mice (black circles; n = 8) showed impairments in acquisition and reversal learning in the Morris water maze test. (**c**) Female *Akt1*^−/−^ mice swam a shorter distance in the target quadrant than female WT controls on the probe test (Day 9). *p < 0.05.

We further investigated whether the observed alterations in hippocampal neural oscillations led to changes in hippocampus-related cognitive functions (Y-maze and Morris water maze task performance) in female *Akt1*^−/−^ mice. In the Y-maze task (Experiment 6a), female *Akt1*^−/−^ mice exhibited impairments in the relative time spent (*t*(14) = 2.075, *p = *0.057) and distance travelled in the novel arm (*t*(14) = 3.082, *p* < 0.05) compared to their WT controls (Fig. 6a). In the Morris water maze test (Experiment 6b), female *Akt1*^−/−^ mice learned less rapidly than their WT controls. Female *Akt1*^−/−^ mice took a significantly longer time to find the platform during the acquisition (*F*(1,14) = 14.711, *p* < 0.05) and reversal trials (*F*(1,14) = 7.141, *p* < 0.05), as shown in Fig. 6b. On the probe trial (Day 9), female *Akt1*^−/−^ mice swam a shorter distance in the target quadrant than their WT controls (*t*(14) = 2.870, *p* < 0.05), but no difference in swimming activity in the other quadrants was found between the groups (Fig. 6c). Thus, it is evident that female *Akt1*^−/−^ mice displayed impairments in hippocampus-related cognitive functions on the Y-maze and Morris water maze tasks.

## Discussion

Guided by the convergent results of human genetic studies, several assays were performed in this study to evaluate the effect of Akt1 deficiency on the ratio of GABA interneurons, GABA_A_R expression, and hippocampus-related cognitive functions *in vitro* and *in vivo*. Inhibiting Akt had no effect on overall neuronal differentiation but resulted in a significant reduction in the proportion of GABAergic neuron-like cells. Compared to female WT controls, female *Akt1*^−/−^ mice (1) exhibited less GABA_A_R antagonist-induced convulsive activity, (2) had fewer parvalbumin-positive interneurons and lower expression of the GABA_A_R β2 subunit, especially in the hippocampus, (3) displayed morphological alterations in the CA1 pyramidal neurons of the dorsal hippocampus, (4) had increased hippocampal oscillation power, and (5) showed impaired cognitive performance on two hippocampus-related tasks.

Accumulating evidence from human studies and animal models indicates that Akt1 is involved in the regulation of dopaminergic signalling and dopamine-associated functions^3^,^4^,^10^,^11^,^15^,^35^,^36^, and this evidence suggests the importance of Akt1 in the dopamine hypothesis of schizophrenia. In contrast, the GABA hypothesis of schizophrenia proposes that reduced neuronal GABA concentrations and GABAergic neurotransmission result in cognitive impairments in schizophrenia. The GABA hypothesis allows for the integration of the GABAergic and oscillatory abnormalities into the glutamate hypothesis of schizophrenia, referred to as the “GABAergic interneuron origin hypothesis of schizophrenia”^17^,^18^,^37^. Intriguingly, in Experiments 1–3 of this study, Akt1 deficiency modulated the numbers of parvalbumin-positive interneurons, the convulsive activity induced by a general GABA_A_R antagonist, and the expression of the GABA_A_R β2 subunit in the hippocampus. These findings are consistent with the current evidence supporting the GABA hypothesis, in which reductions in the subpopulation of GABAergic interneurons expressing parvalbumin and the GABA_A_R β2 subunit have been reported in animal models of schizophrenia and in the post-mortem brains of schizophrenia patients^18^,^19^,^20^,^21^,^22^. Our findings also support the involvement of Akt1 in the modulation of parvalbumin-positive interneurons and GABAergic signalling, thereby implicating Akt1 in the GABA hypothesis of schizophrenia.

Importantly, the inhibitory activity of parvalbumin-positive interneurons plays a crucial role in generating synchronous gamma oscillations^34^,^37^, and accumulating evidence implicates disturbed neuronal synchrony in the gamma frequency range as an important physiological feature of schizophrenia^38^. It was interesting to observe a specific reduction in the abundance of hippocampal parvalbumin-positive (but not calretinin-positive) interneurons in Experiment 3 of our study, and the difference in the consequences of *Akt1* deficiency between parvalbumin-positive and calretinin-positive interneurons may be because these cell types differentiate from different origins of hippocampal neurogenesis during development^39^. In addition to our observed reductions in the number of hippocampal parvalbumin-positive interneurons and in GABA_A_R expression in association with *Akt1* deficiency, we demonstrated in Experiment 5 that anaesthetized *Akt1*-deficient mice had increased hippocampal oscillation power in the theta, alpha, beta, and gamma frequency ranges. This alteration in hippocampal neural oscillations might contribute, at least in part, to the impairments in hippocampus-associated cognitive function observed in our final experiment. Brain oscillations in the gamma range (30–80 Hz), which are generated by fast-spiking parvalbumin-positive interneurons, have been proposed to play a crucial role in many cognitive functions^40^,^41^. Consequently, disruption of parvalbumin-expressing interneurons is associated with many mental illnesses, including schizophrenia^37^,^42^. Supporting findings from studies of schizophrenia patients indicated that these individuals also have deficits in gamma oscillations^27^. These findings suggest that dysregulation of GABAergic signalling could result in dysfunction of gamma oscillation activity and could consequently lead to the cognitive deficits observed in schizophrenia. Intriguingly, previous preclinical and human studies have commonly shown that deficits in parvalbumin-positive GABAergic neurons result in reduced activity in the gamma frequency range; such findings are somewhat inconsistent with our current finding in *Akt1*^−/−^ mice and certain findings regarding the baseline oscillation power of schizophrenia patients^43^,^44^,^45^. In the current study, local field potentials were recorded in mice under anaesthesia, somewhat similar to the resting state in humans. A recent study also demonstrated increased resting-state connectivity in the gamma frequency range among individuals with first-episode schizophrenia compared to healthy controls^46^. Given that the precise roles of gamma oscillations under different states (e.g., resting state vs. conducting cognitive task) remain unclear, *in vivo* recording of the brains of behaving *Akt1*^−/−^ mice during a cognitive task would be warranted in a future study.

In addition, it is well characterized that synaptic inhibition in the brain is largely mediated by GABA and that the fast inhibitory effects of GABA are mediated by GABA_A_R activation. Intriguingly, Chen and colleagues from our group recently reported that Akt1 deficiency significantly affected the intrinsic electrophysiological properties (including enhanced input resistance, decreased rheobase, increased firing frequency, and reduced GABA_A_R-mediated miniature inhibitory postsynaptic currents) of striatal medium spiny neurons (the principal neurons forming the predominantly GABAergic microcircuit in the striatum) in *Akt1*^−/−^ mutant mice^12^; their findings suggest downregulation of functional GABA_A_R expression or activity in the striatum of *Akt1*-deficient mice. Complementary to our findings in P19 cells (Experiment 1), it was reported that active Akt can promote the differentiation of neuronal precursor cells into GABAergic neuron-like cells^29^ and that phosphorylation of GABA_A_R via insulin-induced Akt signalling can lead to an increase in the number of GABA receptors on the plasma membrane^28^, thereby increasing inhibitory fast synaptic transmission in neurons. Accordingly, as demonstrated in this study, Akt1 deficiency might contribute to the reductions in functional GABA_A_R expression and GABAergic interneuron number in the hippocampus, thereby decreasing inhibitory transmission and altering hippocampal oscillations.

Further, as shown in Experiment 4 of this study, Akt1 deficiency not only affected neural activity in the hippocampus but also altered the morphological features of hippocampal CA1 pyramidal neurons. Similar to pyramidal neurons in other cortical areas, CA1 hippocampal pyramidal neurons primarily receive glutamatergic and GABAergic synaptic inputs. These GABAergic inputs, primarily originating from local interneurons, control the firing rate of the pyramidal neurons, modulate their spike timing, and synchronize their activity^33^. GABAergic interneurons (especially parvalbumin-expressing interneurons) predominantly innervate the dendrites of hippocampal CA1 pyramidal neurons and actively contribute to input/output signalling, signal integration, and synaptic plasticity of these neurons^32^,^33^. It is expected that dendritic GABAergic inputs regulate these dendritic signals in pyramidal neurons. As reported in Experiment 4, significant reductions in the morphological features and complexity of hippocampal CA1 pyramidal neurons, especially in the basal dendrites, were observed in Akt1-deficient mice. Moreover, it was reported that Akt1 might be an important regulator of neurite outgrowth^47^. Further evidence indicates that genetic deletion of Akt1 in mice resulted in a reduction in the morphological complexity of striatal medium spiny neurons^12^ and of pyramidal neurons in the medial prefrontal cortex^10^ and the auditory cortex^11^. Thus, our current finding in the hippocampus is consistent with these previous findings and validates the effect of Akt1 on neuronal morphology. Importantly, the observed alterations in the number of hippocampal parvalbumin-positive interneurons (Experiment 3) and neuromorphological features of pyramidal neurons (Experiment 4) in *Akt1*-deficent mice could contribute to the shift in the balance between excitation and inhibition in the hippocampus. A proper excitation/inhibition balance is critical for the control of hippocampal oscillations and output^32^,^33^. Although we did not concentrate on characterizing electrophysiological properties or excitatory glutamatergic transmission in this study, it was very interesting to find that female *Akt1*-deficient mice exhibited less general GABA_A_R antagonist-induced convulsive activity (Experiment 2) and had increased hippocampal oscillation power (Experiment 5). In future studies, further examination of the electrophysiological properties of neurons in hippocampal slices and of hippocampal neural activity in behaving Akt1 mutant mice would be warranted to elucidate the exact mechanism underlying the imbalance between excitation and inhibition in the hippocampus of these mice.

Furthermore, it was reported that mixed-sex *Akt1*^−/−^ mice exhibited reduced hippocampal neurogenesis and impairments in hippocampus-dependent contextual fear conditioning and recall of spatial memory^31^. Our current behavioural data are highly consistent with those previous behavioural results regarding hippocampus-dependent functions. We further demonstrated that female *Akt1*^−/−^ mice had a spatial memory deficit in the Y-maze task and impaired spatial learning, recall, and reversal in the Morris water maze task. One possible explanation for the observed hippocampus-dependent deficits is that Akt1 deficiency affects the morphological features of hippocampal CA1 pyramidal neurons and the abundance of local GABAergic interneurons, resulting in alterations in the firing rate of pyramidal neurons and in the synchronicity of neural activity, as described above. Somewhat surprisingly, we found that female *Akt1*^−/−^ mice, but not male *Akt1*^−/−^ mice, are less sensitive to PTZ-induced seizures than their WT controls. Accordingly, we simply focused on female mice in the remainder of our experiments due to time constraints and manpower limitations. However, because we did not conduct all experiments on both males and females, the potential involvement of Akt1 in the regulation of GABAergic interneurons and hippocampal oscillations in males cannot be completely ruled out. Given that the sample size of male mice is somewhat small and that there was a non-significant trend toward reduced severity of PTZ-induced seizures among male *Akt1*^−/−^ mice compared to male WT mice, caution should be taken when interpreting the sex differences in PTZ-induced convulsions and GABAergic functions observed in the current study. Nonetheless, sex-specific effects of Akt1 deficiency on behavioural phenotypes have been well characterized in Akt1-deficient mice in previous studies^11^,^12^. Numerous sex-based differences in the risk of schizophrenia have also been reported in humans^48^,^49^, and a sex-based difference in the association of the *Akt1* gene with the risk of schizophrenia has been reported, as well^50^. The mechanisms underlying the sex-specific effect of Akt1 and the effects of hormones (e.g., oestrogen) on GABAergic transmission and the pathogenesis of schizophrenia merit further investigation in future studies.

It is also of great interest to link some of current results to the findings in humans or patients with schizophrenia. Indeed, several studies have reported on the effect of Akt1 on hippocampal function, structure, or postmortem expression in humans, and the reported results are somewhat consistent with our current findings. First, lower Akt1 protein levels were reported in the hippocampus of patients with schizophrenia^4^. Second, reduced levels of Akt phosphorylated at serine 473 were reported in hilar neurons of the dentate gyrus in postmortem brains of patients with schizophrenia compared with healthy controls^31^. Third, first-episode schizophrenia patients who did not receive psychotropic medications displayed a decreased Akt phosphorylation ratio in B lymphocytes and exhibited reduced left hippocampal volume compared with healthy controls^51^. Fourth, the Akt1 rs1130233 variant was linked to differential Akt1 protein expression levels in human lymphoblasts^15^ and influenced gray matter volume in the medial temporal lobe as well as memory-dependent hippocampal activity^52^. Taken together, the findings of this study indicate the involvement of Akt1 in the regulation of hippocampal GABAergic interneuron abundance, functional GABA_A_R expression, neuronal morphology, neuronal activity oscillations, and hippocampal-dependent cognitive functions in a mouse model of schizophrenia. These results indicate the contribution of Akt1 to the pathogenesis of schizophrenia and support the GABA hypothesis of schizophrenia.

## Materials and Methods

### P19 cell culture

P19 mouse embryonal carcinoma cells (P19 cells) were selected because neuron-like cells derived from P19 cells express glutamic acid decarboxylase^53^ and multiple *GABA*_*A*_*R* subunits^54^. P19 cells were maintained in αMEM with supplements^55^.

### Animals

All adult (2–4-month-old) *Akt1*^−/−^ and WT mice used in this study were generated from *Akt1*^+/−^ breeding pairs in the C57BL/6 genetic background (*n* > 10). All animals were 2–3 months old at the beginning of the experiments. The details of the animal experiments have been described elsewhere^11^,^12^ and in the supplementary materials. All animal procedures were performed according to protocols approved by the appropriate Animal Care and Use Committees established by National Taiwan University. The minimum number of mice was used in accordance with the 3R principle of animal use. Adequate measures were utilized to minimize potential pain or discomfort experienced by the mice used in this study.

### Experiment 1: The effect of Akt1/2 inhibitor application on neuronal differentiation and the production of GABAergic neuron-like cells from P19 cells

For neuronal differentiation, P19 cells were transfected with the US2-Ascl1 vector to induce neural differentiation, as described elsewhere^56^. An Akt1/2 inhibitor (1 μM, Calbiochem, San Diego, CA) was mixed into the medium during DIV 0–5. To measure the differentiation of GABAergic neuron-like cells derived from P19 cells, immunocytochemistry was performed at DIV 5 using antibodies against GFP (for transfected cells), Tuj1 (for differentiated neurons), GAD67 (for GABAergic interneurons), and parvalbumin (for parvalbumin-positive neurons). The proportion of neuron-like cells, GABAergic neuron-like cells, and parvalbumin-positive neuron-like cells derived from P19 cells are shown as the proportions of cells expressing Tuj1, GAD67, or parvalbumin among all GFP-positive cells.

To examine functional GABA_A_R expression, P19 cells were transfected with the US2-puro and US2-Ascl1 vectors. After selecting and extracting the transfected cells, Western blotting was performed using an antibody against the GABA_A_R β2 subunit and an antibody against the Na^+^/K^+^ ATPase as a loading control. Densitometric analysis was performed using NIH ImageJ software. Details are presented in the supplementary methods.

### Experiment 2: General evaluation of PTZ-induced seizures in male and female *Akt1*-deficient mice

PTZ (Sigma-Aldrich, St. Louis, MO) was administered to induce seizures for examination of potential abnormalities in GABA-related phenotypes in both male and female *Akt1*^−/−^ mice compared to their WT littermates (n = 6 for the male groups; n = 8 for the female groups). PTZ dissolved in saline was administered subcutaneously at a dose of 50 mg/kg (5 mg/ml). After injection, animal behaviours were videotaped immediately for up to 30 min in a clean cage with bedding. The following two behavioural indexes adapted from a previous study^57^ were used. (A) Latency: the latency (sec) to the initial onset of seizures was recorded. (B) Severity: The severity of seizure activity was scored blindly using the following scale: 0, no signs of motor seizures; 1, isolated twitches; 2, tonic-clonic convulsions; 3, tonic extension; and 4, death.

### Experiment 3: Examination of the GABAergic interneuron density and the GABA_A_R expression level in the brains of female *Akt1* ^−/−^ mice

Because GABA is mediates pre- and post-synaptic inhibition of neuronal activity, two sub-experiments were designed to examine the number of GABAergic interneurons and the level of functional GABA_A_R expression in the target brain regions of female mice based on the results of Experiments 1 and 2. Experimental details are provided in the supplementary methods.

#### Experiment 3a: Examination of the density of GABAergic interneurons in the brains of female mice

Immunohistochemistry was conducted on brain sections from female *Akt1*^−/−^ mice (*n = *6) and their WT littermates (*n = *8) to label two major subtypes of GABAergic interneurons using antibodies against parvalbumin and calretinin. Neuronal density in the CA1 and CA3 subregions of the hippocampus and in the cortices (anterior cingulate cortex area 1 (aCg1), prelimbic cortex (PrL), infralimbic cortex (IL), primary motor cortex (M1), and primary auditory cortex (Au1)) was measured using NIH ImageJ software.

#### Experiment 3b: Examination of the expression of functional GABA_A_ receptors in the target brain areas of female mice

Based on previous findings and our results from Experiments 1 and 2, Western blotting was utilized to examine the functional expression of GABA_A_Rs in the hippocampus, cortex, and striatum of adult female *Akt1*^−/−^ mice and their WT littermates (n = 5 each). The membrane fraction of brain lysates was extracted using a commercial kit (Fermentas), and the expression levels of GABA_A_R and Na^+^/K^+^ ATPase were examined as described for Experiment 1.

### Experiment 4: Examination of the morphological features of hippocampal pyramidal neurons in female *Akt1* ^−/−^ mice

Based on the findings from Experiments 2 and 3, additional female subjects generated from *Akt1*^+/−^ breeding pairs in a C57BL/6-*Tg* (GFPm) background were used to investigate neuronal morphology. Morphometric analysis of GFP-labelled pyramidal neurons in the CA1 region of the dorsal hippocampus of adult female *Akt1*^−/−^ (n = 13) and WT (n = 11) mice was conducted to detect genotypic differences in neuronal morphology. For each complete and available neuron in the CA1 area, 10 morphological variables that were selected based on previous studies^11^,^58^ were examined. Details are provided in the supplementary methods.

### Experiment 5: Recording of neuronal oscillations in the hippocampus of anaesthetized female mice

Hippocampal oscillatory activity was examined to further assess whether *Akt1* deficiency alters neuronal activity in the hippocampus. Adult female *Akt1*^−/−^ mice and WT littermate controls (n = 5 each) were used, and electrodes were implanted into the CA1 region of the dorsal hippocampus. Details of electrode implantation and histology are provided in the supplementary methods. After recovery, the mice were anaesthetized with isoflurane (1%), and local field potentials (LFPs) were recorded from the hippocampus for 30 min using a Plexon system (Plexon Inc., Dallas, TX). Spectral analysis of LFP power was performed using NeuroExplorer (Plexon Inc.). The power spectra were calculated using Welch’s method (512 frequencies between 1 and 100 Hz, smoothed using a Gaussian Kernel with a bin width of 3). The mean oscillation power was averaged for different frequency ranges (delta: 0–4 Hz; theta: 5–8 Hz; alpha: 9–12 Hz; beta: 12–30 Hz; and gamma: 30–80 Hz).

### Experiment 6: Examination of hippocampus-related cognitive function in female *Akt1* ^−/−^ mice

Based on the results from the above experiments and previous studies of *Akt1*^−/−^ mice^11^,^12^,^31^, two sub-experiments were conducted to examine the potential genotypic differences in hippocampus-related cognitive function in female *Akt1*^−/−^ mice compared to female WT mice. Details are provided in the supplementary methods.

#### Experiment 6a: Examination of spatial memory using the Y-maze task

The retention of spatial memory by naïve female *Akt1*^−/−^ mice and WT littermates (n = 8 each) was examined using a Y-maze task. The Y-maze test consisted of a 10-min training trial and a 5-min retention trial separated by a one-hour inter-trial interval. Details of the experimental procedure are provided in the supplementary methods. The following two indices of spatial memory retention were calculated: (1) the proportion of time spent in the novel arm and (2) the relative distance travelled in the novel arm.

#### Experiment 6b: Examination of spatial learning and memory using the Morris water maze task

Spatial learning and memory were further examined using a standard spatial version (hidden platform) of the Morris water maze task (n = 8 per group). Details are provided in the supplementary methods. Briefly, to assess the acquisition and retention of spatial memory, 6 swimming trials per day (with inter-trial intervals of 11–15 min) were performed for 8 consecutive acquisition days. The escape latency (sec) to reach the hidden platform and the path length (cm) were recorded. After 8 days of training, each subject was returned to the pool without a platform for a 1-min probe test on Day 9. The time spent swimming and the swimming distance in each quadrant were recorded. One day after the probe test, each mouse was retrained in the reversal version of the water maze for 5 consecutive days to test reversal learning.

## Statistics and Data Analyses

All data are presented as the means ± standard error of the mean (SEM). The data that met the assumptions for normality and homogeneity of variance based on Kolmogorov–Smirnov tests were analysed using parametric tests. All of the data were normally distributed (data not shown). As appropriate, statistical evaluations were performed using Student’s *t*-test or ANOVA to detect genotypic differences using SPSS 20.0 (SPSS Inc., Chicago, IL, U.S.A.). *Post hoc* analysis was performed using Fisher’s LSD test when the *F* values indicated significant differences between groups, and *p* values of < 0.05 were considered statistically significant.

## Additional Information

**How to cite this article**: Chang, C.-Y. *et al*. Akting up in the GABA hypothesis of schizophrenia: Akt1 deficiency modulates GABAergic functions and hippocampus-dependent functions. *Sci. Rep.* **6**, 33095; doi: 10.1038/srep33095 (2016).

## Supplementary Material

Supplementary Information
