# Supplementary material for: Akting up in the GABA hypothesis of schizophrenia: Akt1 deficiency modulates GABAergic functions and hippocampus-dependent functions
Source: Sci Rep. 2016 Sep 12;6:33095. doi: 10.1038/srep33095 (PMC5018883; doi:10.1038/srep33095)
Supplement: Supplementary Information [file srep33095-s1.pdf]

Supplementary Methods and Supplementary Figures:

MS Title: Akting up in the GABA hypothesis of schizophrenia: Akt1 deficiency modulates GABAergic functions and hippocampus-dependent functions

Author List: Chia-Yuan Chang [1], Yi-Wen Chen [1], Tsu-Wei Wang [2], Wen-Sung Lai [1, 3, 4]

[1] Department of Psychology, National Taiwan University, Taipei, Taiwan

[2] Department of Life Science, National Taiwan Normal University, Taipei, Taiwan

[3] Graduate Institute of Brain and Mind Sciences, National Taiwan University, Taipei, Taiwan

[4] Neurobiology and Cognitive Science Center, National Taiwan University, Taipei, Taiwan

Supplementary Methods

Animals:

All *Akt1*$^{-/-}$ and WT mice used in this study were generated from *Akt1*$^{+/-}$ breeding pairs in the C57BL/6 genetic background (backcrossed for more than 10 generations) and were genotyped via PCR analysis of mouse-tail DNA, as described previously[1-3]. After weaning, animals were housed in ventilated polysulfone cages (Alternative Design Manufacturing & Supply, Arkansas, AR, U.S.A.) within the animal rooms of

the Psychology Department, National Taiwan University, and provided with food and water *ad libitum*. All animals were 2–3 months old at the beginning of the experiments. Animals were handled and weighed daily beginning at least 1 week before the behavioural experiments. All animal procedures were performed according to protocols approved by the appropriate Animal Care and Use Committees established by National Taiwan University. The minimum number of mice was used in accordance with the 3R principle of animal use. Adequate measures were taken to minimize potential pain or discomfort experienced by the mice used in this study.

Experiment 1: The effect of an Akt inhibitor on neuronal differentiation and the abundance of GABAergic neuron-like cells from P19 cells

For neuronal differentiation, P19 mouse embryonal carcinoma cells were further split 16 hours after transfection into dishes coated with 2 μg/ml mouse natural laminin (Invitrogen, Carlsbad, CA, U.S.A.) and cultured in Opti-MEM1 medium supplemented with 1% FBS. P19 cells were transfected with US2-Ascl1 to induce neuronal differentiation, as described elsewhere[4]. The Akt1/2 inhibitor (1 μM, Calbiochem, San Diego, CA, U.S.A.) was mixed into the medium during DIV 0-5. To measure the differentiation of GABAergic neuron-like cells from P19 cells, the P19 cells were transfected with 0.5 μg of US2-GFP (control plasmid for transfection) and 0.5 μg of

US2-Ascl1 (inducer of neuronal differentiation). To examine the expression of GABAergic neuronal markers, immunocytochemistry was performed at DIV 5 to label transfected cells (anti-GFP antibody; 1:2000, Molecular Probes, Eugene, OR, U.S.A.), differentiated neurons (anti-Tuj1 antibody; 1:1000, Covance Inc., Princeton, NJ, U.S.A.), and GABAergic interneurons (anti-GAD67 antibody; 1:500, EMD Millipore, Billerica, MA, U.S.A.). We also evaluated the two major subtypes of GABAergic interneurons using antibodies against calretinin (1:2000, EMD Millipore) and parvalbumin (1:1500, Sigma-Aldrich Co., St. Louis, MO, U.S.A.). The cells were first fixed in 4% paraformaldehyde (PFA) and washed with PBS containing 0.2% Triton (PBT). Then, the cells were incubated overnight at 4 °C with primary antibodies diluted in 5% goat serum and 0.5% Triton in PBS. After washing, the cells were incubated with secondary antibodies at room temperature for 2 hours. Neuronal density was quantified using ImageJ software (NIH, Bethesda, MD, U.S.A.). Microscopic images of stained neurons were obtained using 20x and 40x objectives on a Nikon Eclipse 80i microscope (Nikon, Tokyo, Japan) with Image-Pro Plus v7.0 software (Media Cybernetics, Rockville, MD, U.S.A.). The percentages of differentiated neuron-like cells, GABAergic neuron-like cells, and parvalbumin-positive neuron-like cells among all P19-derived cells are shown as the percentages of GFP-positive cells that were also positive for Tuj1, GAD67, or parvalbumin, respectively.

Regarding functional $GABA_AR$ expression, previous studies have reported that all functional $GABA_ARs$ require a β subunit[5] and that the phosphorylation of the $GABA_AR$ β2 subunit via Akt plays a crucial role in the efficacy of $GABA_AR$-mediated synaptic inhibition both *in vitro* and *in vivo*[6]. P19 cells were first transfected with 1.25 μg of US2-puromycin (for selection of transfected cells) and 1.25 μg of US2-Ascl1. Transfected cells were selected using 15 μg/ml puromycin. Next, the cells were washed twice with PBS and lysed with a commercial permeabilization buffer (ProteoJET™ Membrane Protein Extraction Kit, K0321, Fermentas, Waltham, MA, U.S.A.) containing Protease Inhibitor Cocktail tablets (Roche, Basel, Switzerland) and Phosphatase Inhibitor Cocktail 1 (Sigma-Aldrich). Then, the mixture was incubated for 10 min at 4 °C with continuous rocking. Afterwards, the mixture was centrifuged at 16,000 g at 4 °C for 15 min. The supernatant, containing cytoplasmic proteins, was carefully collected for subsequent analysis. The cell debris was placed on ice, and commercial membrane protein extraction buffer was added. The debris was homogenized, and the mixture was then shaken at 1400 rpm for 30 min at 4 °C. Afterwards, the mixture was centrifuged at 16,000 g at 4 °C for 15 min. The supernatant was collected, and the protein concentration was measured using the Bradford protein assay (Bio-Rad Laboratories, Tokyo, Japan) via spectrometry at 620 nm. Protein samples were separated via sodium dodecyl sulfate-polyacrylamide gel electrophoresis

(SDS-PAGE) on 10% polyacrylamide gels and transferred onto nitrocellulose membranes (EMD Millipore). A mouse anti-β2 $GABA_A$R subunit antibody (1:1000, EMD Millipore) was used as the primary antibody, and a rabbit anti-Na^+/K^+ ATPase antibody (1:1000, Cell Signaling Technology, Danvers, MA, U.S.A.) was used as the loading control of membrane protein. Appropriate HRP-conjugated antibodies were used as secondary antibodies. Bound antibody was detected using an enhanced chemiluminescence kit (EMD Millipore) and blue sensitive universal film (Fujifilm, Tokyo, Japan). Densitometric analysis was performed using NIH ImageJ software.

Experiment 3: Examination of the numbers of GABAergic interneurons and of $GABA_A$R expression in the brains of female *Akt1*$^{-/-}$ mice and female WT littermate controls.

Because GABA is mediates pre- and post-synaptic inhibition of neuronal activity, two sub-experiments were designed to examine the numbers of GABAergic interneurons and the expression of $GABA_A$Rs in the target brain regions of female mice based on the results of Experiments 1 and 2.

Experiment 3a: Examination of the expression of GABAergic interneurons in the brains of female mice. Animals were perfused with 0.1% saline followed by 4% PFA. The mouse brains were serially sectioned using a cryostat (HM-520, Thermo

Scientific, Waltham, MA, U.S.A.) at 40 μm per section. The sections were first incubated in 3% H_2O_2 to block endogenous peroxidase activity. After washing with 0.02 M KPBS (pH = 7.0), the sections were blocked in 5% (w/v) skim milk for 1 hour at room temperature. Then, the sections were incubated in 5% skim milk containing the primary antibody overnight at 4 °C. Immunohistochemistry was conducted on brain sections from female *Akt1*$^{-/-}$ mice (n = 6) and female WT littermates (n = 8) to detect two major subtypes of GABAergic interneurons using antibodies against parvalbumin (1:1500, Sigma-Aldrich) and calretinin (1:2000, EMD Millipore). After incubation in the appropriate secondary antibodies, bound antibodies were detected using DAB substrate (D5905, Sigma-Aldrich) and a Vectastain ABC kit (PK-6100, Vector Laboratories, Burlingame, CA, U.S.A.). Neuronal density was measured in subregions of the hippocampus (CA1 and CA3 areas) and the cortex (anterior cingulate cortex area 1 (aCg1), prelimbic cortex (PrL), infralimbic cortex (IL), primary motor cortex (M1), and primary auditory cortex (Au1)) using NIH ImageJ software.

Experiment 3b: Examination of functional $GABA_A$R expression in the target brain areas of female mice. The expression of $GABA_A$Rs was examined in the brains of adult female *Akt1*$^{-/-}$ and WT mice (n = 5 each) using Western blotting. Brain regions including the hippocampus, whole cortex, and striatum were rapidly dissected, frozen in liquid nitrogen, and stored at -80 °C until protein extraction. Tissue samples were

homogenized in a commercial permeabilization buffer (ProteoJET^{TM} Membrane Protein Extraction Kit, K0321, Fermentas) containing Protease Inhibitor Cocktail tablets (Roche) and Phosphatase Inhibitor Cocktail 1 (Sigma-Aldrich), and the mixture was then incubated for 10 min at 4 °C with continuous rocking. Afterwards, the mixture was centrifuged at 16,000 g at 4 °C for 15 min. The supernatant, containing cytoplasmic proteins, was carefully collected for subsequent analysis. The cell debris was placed on ice and treated with a commercial membrane protein extraction buffer. The debris was homogenized, and the mixture was then shaken at 1400 rpm for 30 min at 4 °C. Afterwards, the mixture was centrifuged at 16,000 g at 4 °C for 15 min. The supernatant was collected, and the protein concentration was measured using the Bradford protein assay (Bio-Rad Laboratories) via spectrometry at 620 nm. Equal amounts of protein were separated via 10% sodium dodecyl sulfate/polyacrylamide gel electrophoresis (SDS/PAGE) and transferred onto nitrocellulose membranes (EMD Millipore) in transfer buffer containing 20% methanol. Following protein transfer, the membranes were washed in Tris-buffered saline containing 0.1% Tween-20 (TBST) and blocked in 5% skim milk for 1 hour at room temperature. Similar to Experiment 1, the membranes were incubated in 5% skim milk containing a mouse anti- β2 $GABA_AR$ subunit antibody (1:1000, EMD Millipore) as the primary antibody and a rabbit anti-Na^+/K^+ ATPase antibody (1:1000, Cell Signaling Technology) as the loading control for

membrane protein. Appropriate HRP-conjugated antibodies were used as secondary antibodies. Bound antibodies were detected using an enhanced chemiluminescence kit (EMD Millipore) and blue sensitive universal film (Fujifilm). Densitometric analysis was performed using NIH ImageJ software.

Experiment 4: Examination of the morphological features of hippocampal pyramidal neurons in female *Akt1*$^{-/-}$ mice and female WT littermate controls.

Based on the findings from Experiments 2 and 3, the morphological properties of hippocampal neurons were examined in adult female *Akt1*$^{-/-}$ mice and WT littermate controls. Because pyramidal neurons of the hippocampal CA1 region are critical for the afferent and efferent connections of the hippocampus, a transgenic C57BL6-Tg (GFPm) mouse line expressing green fluorescent protein (GFP) driven by the Thy1 promoter was selected and used for morphometric analysis of GFP-labelled CA1 pyramidal neurons in the hippocampus[7]. The expression patterns of GFP-labelled pyramidal neurons in the Thy1-C57BL6-Tg (GFPm) transgenic mouse line have been reported previously[7], and this mouse line has been successfully used to analyse the morphological alterations in pyramidal neurons of mutant mice. Additional female mice generated from *Akt1*$^{+/-}$ breeding pairs in the C57BL6-Tg (GFPm) background were used in this experiment. Adult mice were anaesthetized and transcardially

perfused with PBS followed by 4% PFA in PBS. Fixed brains were sectioned coronally using a vibratome. Serial coronal sections (150 µm in thickness) were collected and mounted on slides for immunofluorescence. Confocal stack images of GFP-labelled neurons were obtained at intervals of 0.4 µm using 20x, 40x-oil and 63x-oil objectives on a Leica TCS SP5 confocal microscopy system (Leica, Wetzlar, Germany). Neurolucida software (Microbrightfield Inc., Williston, VT, U.S.A.) was used to trace and reconstruct the neurons in 3 dimensions. GFP labelling was nearly exclusively restricted to the cell bodies and dendritic trees of CA1 pyramidal neurons of both *Akt1*$^{-/-}$and WT mice. Using Neurolucida software, morphometric analyses of the GFP-labelled pyramidal neurons (1.46 to 2.30 mm posterior to Bregma) of adult female *Akt1*$^{-/-}$ (n = 13) and WT littermate controls (n = 11) were performed to detect the neuromorphological differences between genotypes. The following 10 morphological variables were selected for analysis based on previous studies[2,8]: (1) the number of branches from the apical branches; (2) the number of apical tips; (3) the total length of the apical tuft, which was calculated as the sum of the lengths of the apical stem and the branches that formed the tuft; (4) the soma size (obtained by outlining the cell somas and automatically calculating the pixel areas in µm^2); (5) the number of primary basal dendrites (excluding apical dendrites and axons); (6) the total length of the primary basal dendrites; (7) the number of branches from the basal branches; (8) the number of

basal tips; (9) the total length of the basal dendrites; and (10) basal dendritic complexity based on Sholl analysis.

Experiment 5: Recording of neuronal oscillations in the hippocampus of female *Akt1*$^{-/-}$ mice and their WT littermate controls.

Stereotaxic brain surgery and histology: The mice were initially anaesthetized with a small dose of isoflurane (5%) administered in an induction chamber. The mice were then placed in a stereotaxic frame such that the nose was in the anaesthesia head-holder supplying isoflurane in O_2 (at ~0.5 to 1 L/min). The initial isoflurane levels after induction of anaesthesia were as high as 2%, and the isoflurane level was lowered over the course of surgery to ~1% when the animal reached a stable plane of anaesthesia. An adequate depth of anaesthesia was verified based on the absence of the toe-pinch reflex. Then, the scalp was shaved and cleaned with Betadine and 70% ethanol before incision. Using a fresh scalpel blade, a single incision through the skin was made. A single burr-hole in the skull at the target site (CA1 area: AP: -1.8 mm; ML: 1.5 mm) was produced using a drill. The dura was opened slightly, and the electrode array was implanted into the brain tissue at the desired angle and depth (DV: 1.5 mm) by slowly lowering the device. A stainless steel screw driven into the skull above the cerebellum, served as ground. After the completion of implantation, the animal was allowed to fully recover in a clean cage supplied with a heating blanket. Each mouse received an analgesic agent

(e.g., ibuprofen) for a few days after surgery. At least 7 days after recovery, neural oscillations were recorded in female *Akt1*$^{-/-}$ mice and WT littermate controls (n = 5 each) anesthetized using isoflurane (1%). One day after completion of the neural recordings, electrode placement was confirmed post-mortem after perfusion and fixation with 4% PFA and 1% potassium ferrocyanide, followed by Prussian blue staining in 40-μm coronal sections.

Experiment 6: Examination of hippocampus-related cognitive function in female *Akt1*$^{-/-}$ mice and female WT littermate controls.

Based on the findings from Experiments 3-5, hippocampus-related cognitive functions were evaluated using the Y-maze (Experiment 6a) and Morris water maze tasks (Experiment 6b).

Experiment 6a: The Y-maze test. Spatial memory retention was examined in naïve female adult *Akt1*$^{-/-}$ mice and WT littermates (n = 8 each) using a polypropylene Y-maze (with three equally spaced arms (50 cm long, 12 cm wide, 16 cm height)) surrounded by visual cues. The Y-maze test consisted of a 10-min training trial and a 5-min retention trial separated by a one-hour inter-trial interval to assess spatial recognition memory. In the training trial, each mouse was allowed to explore only two arms (the starting arm and another arm) of the maze, with the third arm (the novel arm) blocked.

In the retention trial, each mouse was free to explore all three arms. The distance travelled and time spent in each arm (excluding the central zone of the Y) were recorded and analysed using an EthoVision tracking system (Noldus Information Technology, Wageningen, Netherlands). Two indexes were calculated for the retention trial: (1) the percentage (%) of time spent in the novel arm = time spent in the novel arm / total time spent in any arm; and (2) the percentage (%) of distance travelled in the novel arm = distance travelled in the novel arm / total distance travelled in any arm.

Experiment 6b: The Morris water maze test. Spatial learning and memory abilities were evaluated using a standard spatial version of the Morris water maze task. A circular pool (diameter, 100 cm) filled with water that had been clouded with nontoxic white paint and that was held at 22 ± 1 °C was placed in the centre of the testing room and surrounded by several visual cues. A platform (12×12 cm^2) was hidden 1 cm beneath the surface of the water. The swim paths of each subject were recorded using an EthoVision tracking system (Noldus Information Technology). Each mouse performed 6 trials per day with inter-trial intervals of 11-15 minutes for 8 consecutive acquisition days. On each daily trial, each mouse started from a quadrant that was pseudorandomly selected from the three quadrants that did not contain the platform. Mice that failed to locate the platform within 1 min were gently guided to the platform, where they remained for 30 sec before being returned to their cages. The escape latency

(sec), measured as the time required to reach the hidden platform, and the path length (cm) were recorded. After 8 days of training, the platform was removed from the pool, and each subject was returned to the pool lacking a platform for a 1-min probe test on Day 9. The time spent swimming and the swimming distance in each quadrant were recorded and used as an index of reference memory ability. One day after the probe test, each mouse was retrained in the reversal version of the Morris water maze task, in which the hidden platform was relocated to the quadrant opposite the original target quadrant for 5 consecutive days to test reversal learning.

References:

1 Lai, W. S. *et al.* Akt1 deficiency affects neuronal morphology and predisposes to abnormalities in prefrontal cortex functioning. *Proceedings of the National Academy of Sciences of the United States of America* **103**, 16906-16911, doi:10.1073/pnas.0604994103 (2006).

2 Chen, Y. W. & Lai, W. S. Behavioral phenotyping of v-akt murine thymoma viral oncogene homolog 1-deficient mice reveals a sex-specific prepulse inhibition deficit in females that can be partially alleviated by glycogen synthase kinase-3 inhibitors but not by antipsychotics. *Neuroscience* **174**, 178-189, doi:10.1016/j.neuroscience.2010.09.056 (2011).

3 Cho, H., Thorvaldsen, J. L., Chu, Q., Feng, F. & Birnbaum, M. J. Akt1/PKBalpha is required for normal growth but dispensable for maintenance of glucose homeostasis in mice. *J Biol Chem* **276**, 38349-38352, doi:10.1074/jbc.C100462200 (2001).

4 Lin, Y. T. *et al.* YAP regulates neuronal differentiation through Sonic hedgehog signaling pathway. *Exp Cell Res* **318**, 1877-1888, doi:10.1016/j.yexcr.2012.05.005 (2012).

5 Wan, Q. *et al.* Recruitment of functional GABA(A) receptors to postsynaptic domains by insulin. *Nature* **388**, 686-690, doi:10.1038/41792 (1997).

6 Wang, Q. *et al.* Control of synaptic strength, a novel function of Akt. *Neuron* **38**, 915-928 (2003).

7 Feng, G. *et al.* Imaging neuronal subsets in transgenic mice expressing multiple spectral variants of GFP. *Neuron* **28**, 41-51 (2000).

8 Pei, J. C., Liu, C. M. & Lai, W. S. Distinct phenotypes of new transmembrane-domain neuregulin 1 mutant mice and the rescue effects of valproate on the observed schizophrenia-related cognitive deficits. *Front Behav Neurosci* **8**, 126, doi:10.3389/fnbeh.2014.00126 (2014).

Figure legends for Supplementary Figures

Figure S1. Representative images for Figure 1a. The effect of Akt1/2 inhibitor application on neuronal differentiation from P19 cells was examined using immunocytochemistry. P19 cells were transfected with US2 (control plasmid for transfection) and Ascl1 (inducer of neuronal differentiation). During neuronal differentiation, P19 cells were treated with the vehicle control (Vehicle) or the Akt inhibitor (Akt1/2 inhibitor). At DIV 5, P19 cells were stained for Tuj1 (neuronal marker, red) and GFP (transfection marker, green).

Figure S2. Representative images for Figure 1b. The effect of Akt1/2 inhibitor application on the differentiation of GABAergic neurons from P19 cells was examined using immunocytochemistry. After treatment with an Akt1/2 inhibitor during neuronal differentiation, P19 cells were stained for GAD67 (GABAergic neuronal marker, red) and GFP (transfection marker, green) at DIV 5.

Figure S3. Representative images for Figure 1c. The effect of Akt1/2 inhibitor application on the differentiation of parvalbumin-positive neurons from P19 cells was examined using immunocytochemistry. After treatment with an Akt1/2 inhibitor during neuronal differentiation, P19 cells were stained for parvalbumin (PV;

red) and GFP (transfection marker, green) at DIV 5.

Supplementary Figures S1, S2, & S3

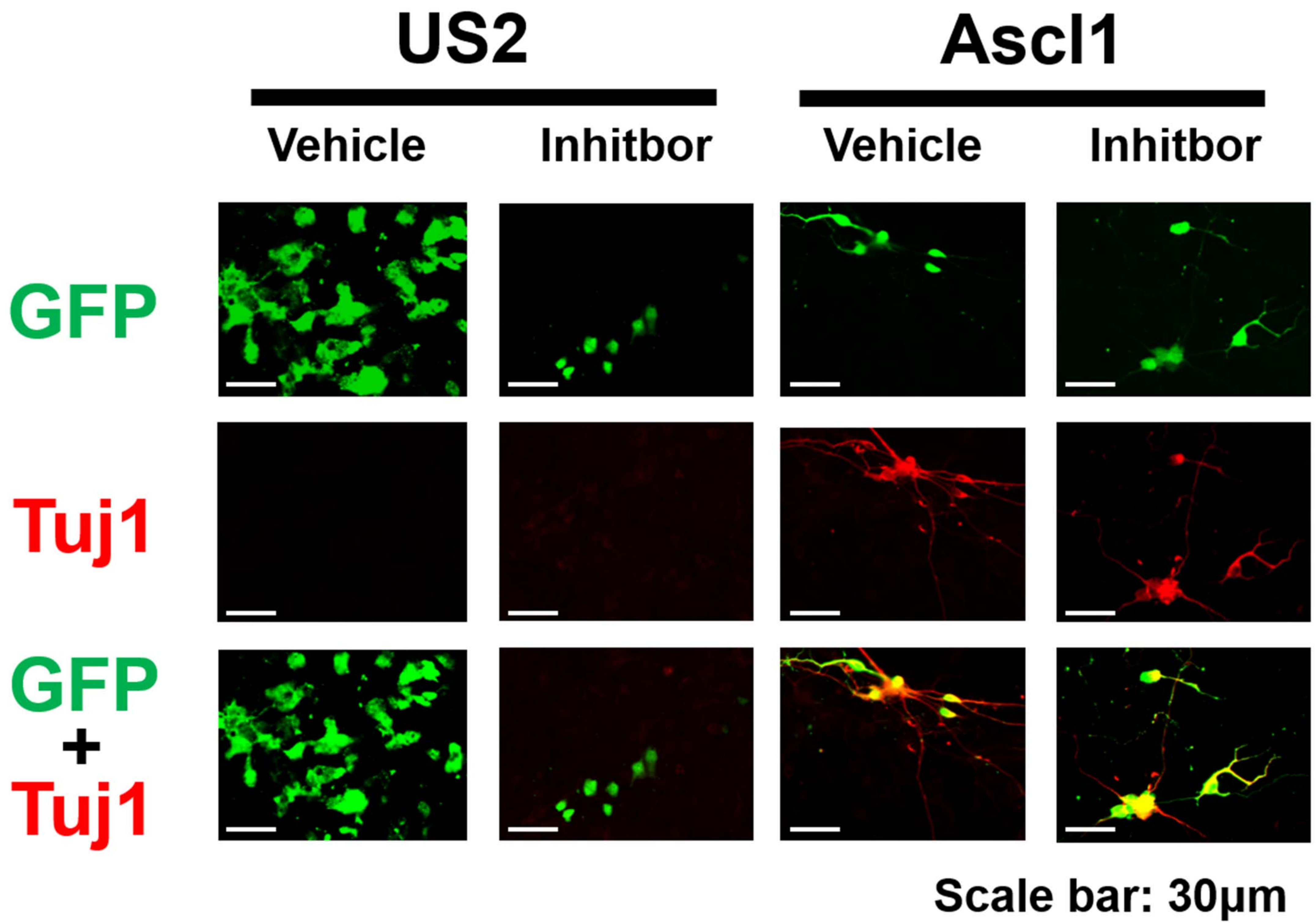
US2
Ascl1
Vehicle
Inhitbor
Vehicle
Inhitbor
GFP
Tuj1
GFP
+
Tuj1
Scale bar: 30μm

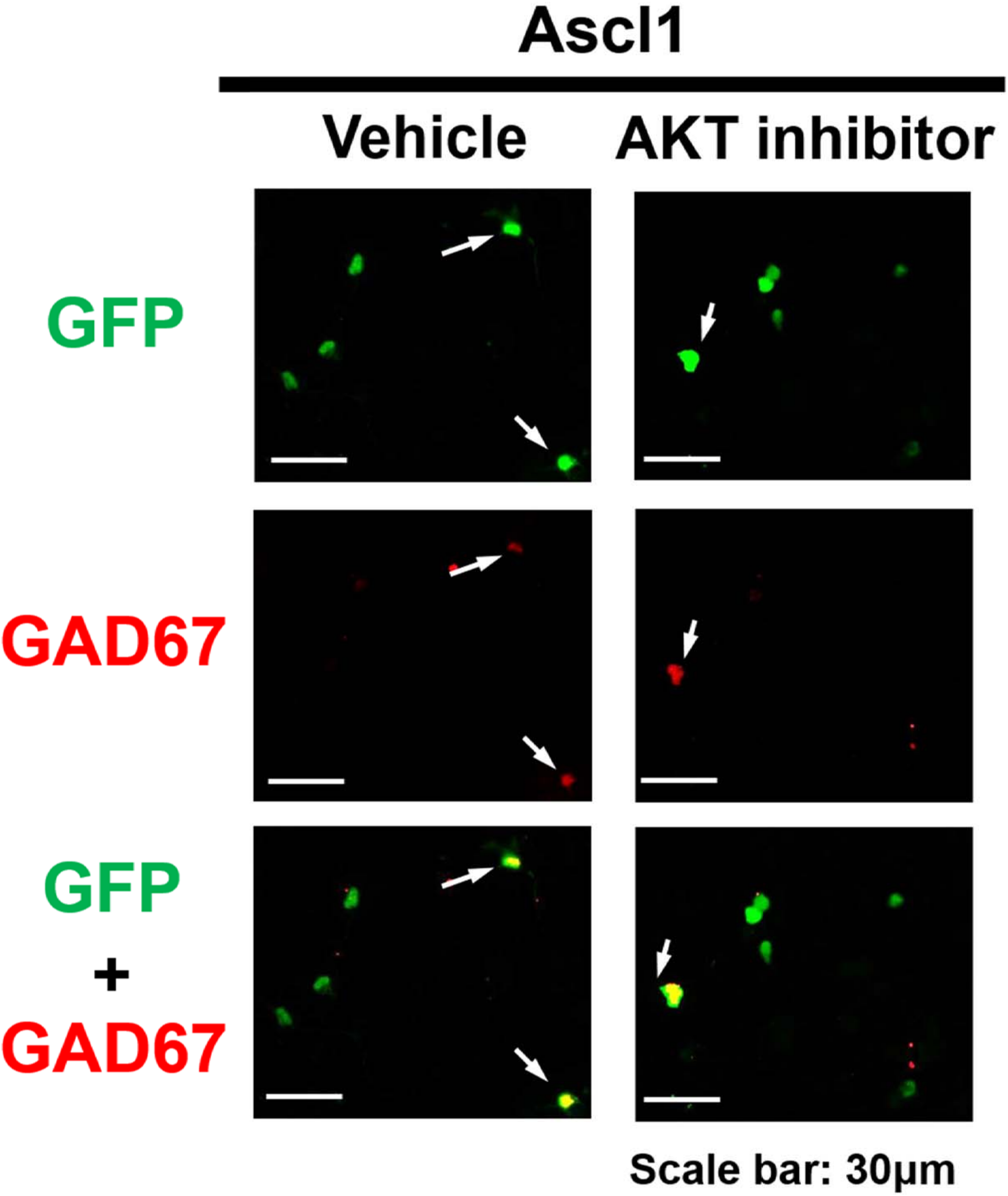
Ascl1
Vehicle
AKT inhibitor
GFP
GAD67
GFP
+
GAD67
Scale bar: 30μm

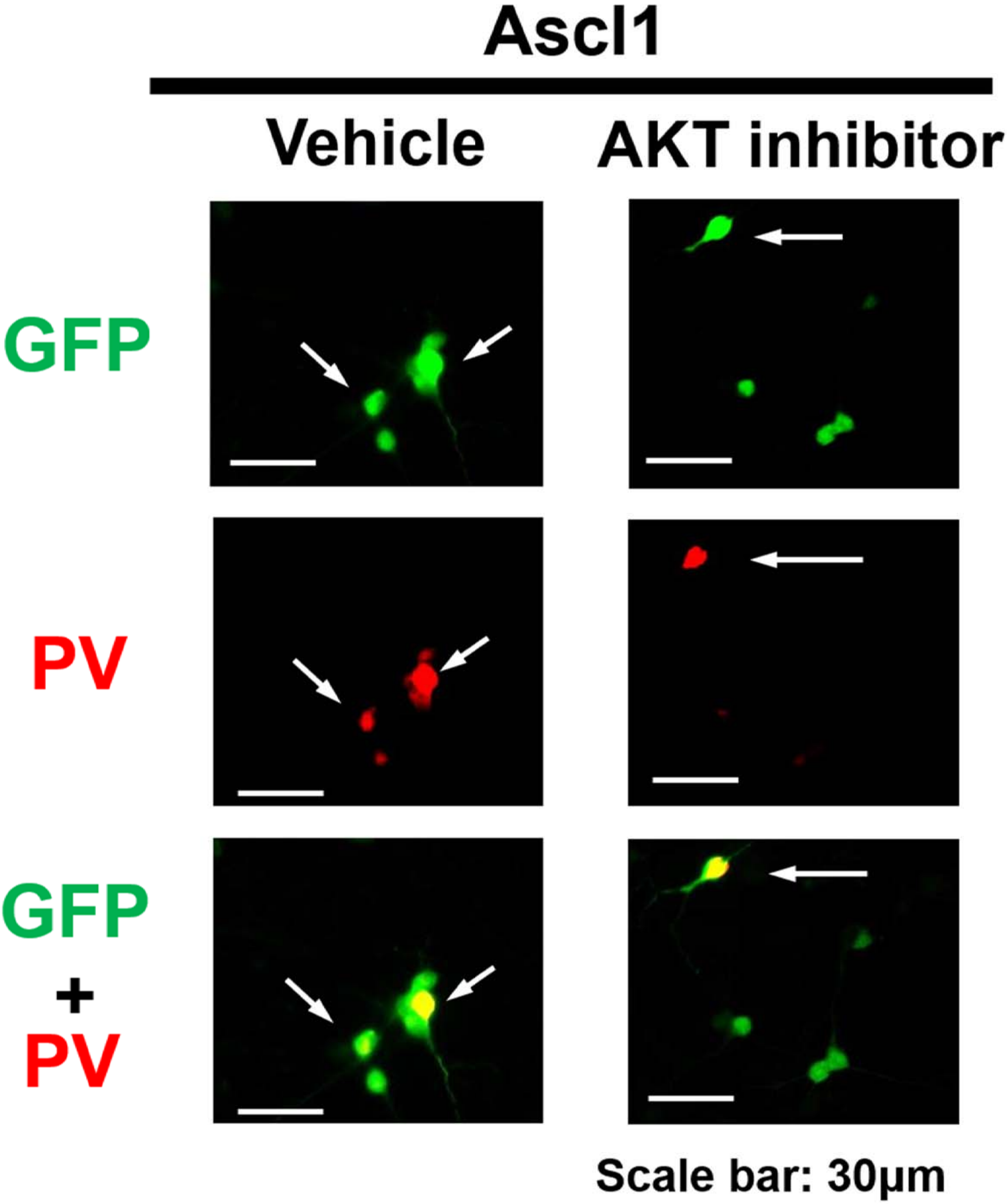
Ascl1
Vehicle
AKT inhibitor
GFP
PV
GFP
+
PV
Scale bar: 30μm
